# Di-μ_2_-ethano­lato-octa­methyl­bis­(μ-4-methyl-5-sulfanyl­idene-4,5-dihydro-1*H*-1,2,4-triazol-1-ido-κ^2^ *N* ^1^:*N* ^2^)di-μ_3_-oxido-tetra­tin(IV)

**DOI:** 10.1107/S1600536812047708

**Published:** 2012-11-28

**Authors:** Ezzatollah Najafi, Mostafa M. Amini, Seik Weng Ng

**Affiliations:** a Department of Chemistry, General Campus, Shahid Beheshti University, Tehran 1983963113, Iran; b Department of Chemistry, University of Malaya, 50603 Kuala Lumpur, Malaysia

## Abstract

The tetra­nuclear title compound, [Sn_4_(CH_3_)_8_(C_2_H_5_O)_2_O_2_(C_3_H_4_N_3_S)_2_], lies about a center of inversion; the mol­ecule features a three-rung-staircase Sn_4_O_4_ core in which two Sn^IV^ atoms are bridged by the 4-methyl-5-sulfanyl­idene-4,5-dihydro-1*H*-1,2,4-triazol-1-ide group. The negatively charged N atom of the group binds to the terminal Sn^IV^ atom at a shorter distance [Sn—N = 2.240 (3) Å] compared with the neutral N atom that binds to the central Sn^IV^ atom [Sn← N = 2.641 (3) Å]. The terminal Sn^IV^ atom is five-coordinate in a *cis*-C_2_SnNO_2_ trigonal–bipyramidal geometry [C—Sn—C = 127.5 (2)°], whereas the central Sn^IV^ atom is six-coordinate in a C_2_SnNO_3_ skew-trazepoidal bipyramidal geometry [C—Sn—C = 145.0 (2)°].

## Related literature

For the [Sn_2_O(CH_3_)_4_(CH_3_O)(C_3_H_4_N_3_S)]_2_ homolog, see: Najafi *et al.* (2011 ▶).
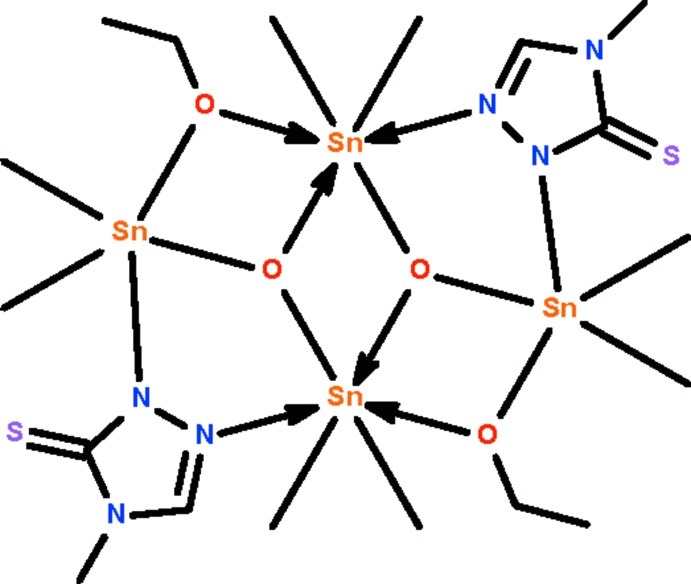


## Experimental

### 

#### Crystal data

[Sn_4_(CH_3_)_8_(C_2_H_5_O)_2_O_2_(C_3_H_4_N_3_S)_2_]
*M* *_r_* = 945.46
Monoclinic, 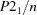

*a* = 9.3965 (4) Å
*b* = 17.8939 (7) Å
*c* = 9.9084 (4) Å
β = 103.036 (4)°
*V* = 1623.06 (11) Å^3^
*Z* = 2
Mo *K*α radiation
μ = 3.20 mm^−1^
*T* = 100 K
0.30 × 0.25 × 0.20 mm

#### Data collection

Agilent SuperNova (Dual, Cu at zero, Atlas) diffractometer
Absorption correction: multi-scan (*CrysAlis PRO*; Agilent, 2012 ▶) *T*_min_ = 0.447, *T*_max_ = 0.567
15728 measured reflections
3743 independent reflections
3280 reflections with *I* > 2σ(*I*)
*R*_int_ = 0.037

#### Refinement

*R*[*F* ^2^ > 2σ(*F* ^2^)] = 0.026
*wR*(*F* ^2^) = 0.065
*S* = 1.04
3743 reflections
155 parameters
H-atom parameters constrained
Δρ_max_ = 0.75 e Å^−3^
Δρ_min_ = −0.67 e Å^−3^

### 

Data collection: *CrysAlis PRO* (Agilent, 2012 ▶); cell refinement: *CrysAlis PRO*; data reduction: *CrysAlis PRO*; program(s) used to solve structure: *SHELXS97* (Sheldrick, 2008 ▶); program(s) used to refine structure: *SHELXL97* (Sheldrick, 2008 ▶); molecular graphics: *X-SEED* (Barbour, 2001 ▶); software used to prepare material for publication: *publCIF* (Westrip, 2010 ▶).

## Supplementary Material

Crystal structure: contains datablock(s) global, I. DOI: 10.1107/S1600536812047708/bt6863sup1.cif

Structure factors: contains datablock(s) I. DOI: 10.1107/S1600536812047708/bt6863Isup2.hkl

Additional supplementary materials:  crystallographic information; 3D view; checkCIF report

## Figures and Tables

**Fig. 1.**
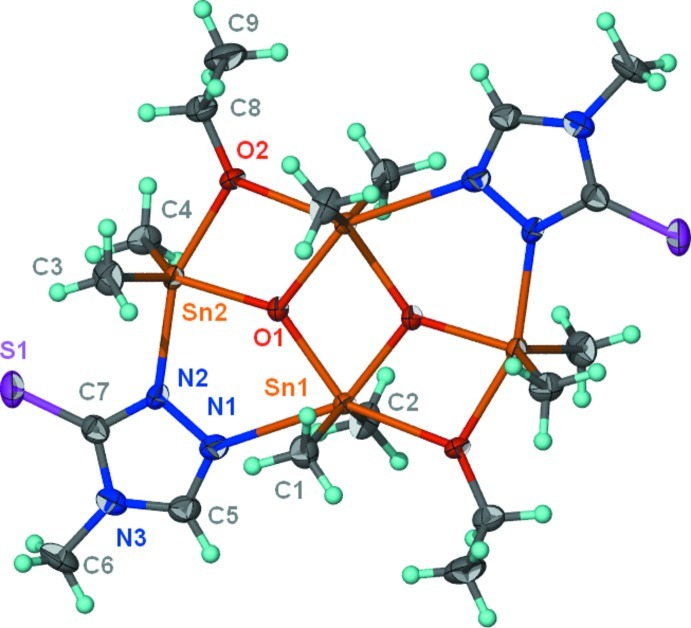
Thermal ellipsoid plot (Barbour, 2001) of Sn4O2(CH3)8(C2H5O)2(C3H4N3S)2 at the 70% probability level; hydrogen atoms are drawn as spheres of arbitrary radius.

## supplementary crystallographic information

### Comment

The title compound (Scheme I, Fig. 1), a distannoxane, was the unexpected product from an attempt at synthesizing a dimethyltin 4-methyl-4*H*-1,2,4-triazol-3-thiolate that possesses a tin-sulfur linkage. In the reaction of diorganotin oxides with organic acids (particularly carboxylic acids), tetranuclear distannoxanes are sometimes formed; these compounds have four organic groups. In the present reaction, two of the four organic groups are replaced by ethoxide groups.

Tetranuclear Sn_4_O_2_(CH_3_)_8_(C_2_H_5_O)_2_(C_3_H_4_N_3_S)_2_ lies about a center-of-inversion; the molecule features a three-rung-staircase Sn_4_O_4_ core in which two Sn atoms are bridged by the C_3_H_4_N_3_S triazolate group. The negatively-charged N atom of the group binds to the terminal Sn atom at a shorter distance [Sn–N 2.240 (3) Å] compared with the neutral N atom that binds to the central Sn atom [Sn←N 2.641 (3) Å]. The terminal Sn atom is five-coordinate in a *cis*-C_3_SnNO trigonal bipyramid whereas the central Sn atom is six-coordinate in a C_2_SnNO_3_ skew-trazepoidal bipyramidal geometry.

### Experimental

Dimethyltin diisothiocyanate (1 mmol), 4-methyl-4*H*-1,2,4-triazole-3-thiol (1 mmol) and 1,10-phenanthroline (1 mmol) were loaded into a convection tube; several drops of triethylamine were added. The tube was filled with dry ethanol and kept at 333 K. Colorless crystals were collected from the side arm after several days.

### Refinement

Carbon-bound H-atoms were placed in calculated positions [C—H 0.95 to 0.98 Å, *U*_iso_(H) 1.2 to 1.5*U*_eq_(C)] and were included in the refinement in the riding model approximation.

### Crystal data

**Table:**

[Sn_4_(CH_3_)_8_(C_2_H_5_O)_2_O_2_(C_3_H_4_N_3_S)_2_]	*F*(000) = 912
*M**_r_* = 945.46	*D*_x_ = 1.935 Mg m^−^^3^
Monoclinic, *P*2_1_/*n*	Mo *K*α radiation, λ = 0.71073 Å
Hall symbol: -P 2yn	Cell parameters from 6388 reflections
*a* = 9.3965 (4) Å	θ = 2.9–27.5°
*b* = 17.8939 (7) Å	µ = 3.20 mm^−^^1^
*c* = 9.9084 (4) Å	*T* = 100 K
β = 103.036 (4)°	Prism, colorless
*V* = 1623.06 (11) Å^3^	0.30 × 0.25 × 0.20 mm
*Z* = 2	

### Data collection

**Table:**

Agilent SuperNova (Dual, Cu at zero, Atlas) diffractometer	3743 independent reflections
Radiation source: SuperNova (Mo) X-ray Source	3280 reflections with *I* > 2σ(*I*)
Mirror monochromator	*R*_int_ = 0.037
Detector resolution: 10.4041 pixels mm^-1^	θ_max_ = 27.6°, θ_min_ = 2.9°
ω scan	*h* = −11→12
Absorption correction: multi-scan (*CrysAlis PRO*; Agilent, 2012)	*k* = −23→21
*T*_min_ = 0.447, *T*_max_ = 0.567	*l* = −11→12
15728 measured reflections	

### Refinement

**Table:**

Refinement on *F*^2^	Primary atom site location: structure-invariant direct methods
Least-squares matrix: full	Secondary atom site location: difference Fourier map
*R*[*F*^2^ > 2σ(*F*^2^)] = 0.026	Hydrogen site location: inferred from neighbouring sites
*wR*(*F*^2^) = 0.065	H-atom parameters constrained
*S* = 1.04	*w* = 1/[σ^2^(*F*_o_^2^) + (0.0302*P*)^2^ + 2.3203*P*] where *P* = (*F*_o_^2^ + 2*F*_c_^2^)/3
3743 reflections	(Δ/σ)_max_ = 0.001
155 parameters	Δρ_max_ = 0.75 e Å^−^^3^
0 restraints	Δρ_min_ = −0.67 e Å^−^^3^

### Fractional atomic coordinates and isotropic or equivalent isotropic displacement parameters (Å^2^)

**Table:**

	*x*	*y*	*z*	*U*_iso_*/*U*_eq_	
Sn1	0.37077 (2)	0.436391 (12)	0.49959 (2)	0.01497 (7)	
Sn2	0.70638 (2)	0.467990 (13)	0.80539 (2)	0.01507 (7)	
S1	0.69104 (11)	0.33447 (6)	1.09067 (10)	0.0342 (2)	
O1	0.5639 (2)	0.48179 (13)	0.6221 (2)	0.0182 (5)	
O2	0.7909 (3)	0.55849 (14)	0.7058 (2)	0.0200 (5)	
N1	0.4168 (3)	0.36557 (17)	0.7393 (3)	0.0224 (6)	
N2	0.5475 (3)	0.37949 (17)	0.8347 (3)	0.0199 (6)	
N3	0.4324 (3)	0.29205 (17)	0.9189 (3)	0.0233 (6)	
C1	0.4547 (4)	0.3342 (2)	0.4438 (4)	0.0253 (8)	
H1A	0.5373	0.3442	0.4012	0.038*	
H1B	0.3782	0.3076	0.3777	0.038*	
H1C	0.4877	0.3034	0.5268	0.038*	
C2	0.2100 (4)	0.4864 (2)	0.5902 (4)	0.0265 (8)	
H2A	0.2210	0.5408	0.5902	0.040*	
H2B	0.2221	0.4686	0.6857	0.040*	
H2C	0.1125	0.4727	0.5366	0.040*	
C3	0.8781 (4)	0.3928 (2)	0.7987 (4)	0.0276 (8)	
H3A	0.9637	0.4208	0.7859	0.041*	
H3B	0.8469	0.3579	0.7214	0.041*	
H3C	0.9034	0.3647	0.8858	0.041*	
C4	0.6810 (4)	0.5344 (2)	0.9736 (3)	0.0247 (8)	
H4A	0.7441	0.5785	0.9803	0.037*	
H4B	0.7081	0.5054	1.0594	0.037*	
H4C	0.5789	0.5504	0.9595	0.037*	
C5	0.3523 (4)	0.3131 (2)	0.7935 (3)	0.0236 (7)	
H5	0.2603	0.2921	0.7505	0.028*	
C6	0.4001 (5)	0.2340 (2)	1.0099 (4)	0.0343 (9)	
H6A	0.3039	0.2124	0.9699	0.051*	
H6B	0.3999	0.2555	1.1008	0.051*	
H6C	0.4747	0.1948	1.0204	0.051*	
C7	0.5556 (4)	0.3356 (2)	0.9450 (3)	0.0212 (7)	
C8	0.9254 (4)	0.5956 (2)	0.7578 (4)	0.0239 (7)	
H8A	0.9841	0.5950	0.6864	0.029*	
H8B	0.9809	0.5685	0.8400	0.029*	
C9	0.9025 (5)	0.6751 (2)	0.7972 (5)	0.0381 (10)	
H9A	0.9974	0.6990	0.8327	0.057*	
H9B	0.8458	0.6758	0.8689	0.057*	
H9C	0.8493	0.7024	0.7155	0.057*	

### Atomic displacement parameters (Å^2^)

**Table:**

	*U*^11^	*U*^22^	*U*^33^	*U*^12^	*U*^13^	*U*^23^
Sn1	0.01475 (12)	0.01498 (13)	0.01399 (11)	−0.00248 (8)	0.00072 (8)	0.00005 (8)
Sn2	0.01482 (12)	0.01626 (13)	0.01265 (11)	0.00072 (8)	−0.00002 (8)	−0.00025 (8)
S1	0.0269 (5)	0.0421 (6)	0.0294 (5)	0.0007 (4)	−0.0028 (4)	0.0170 (4)
O1	0.0186 (12)	0.0190 (12)	0.0146 (11)	−0.0030 (10)	−0.0011 (9)	0.0042 (9)
O2	0.0177 (12)	0.0203 (13)	0.0190 (12)	−0.0075 (10)	−0.0018 (9)	0.0009 (9)
N1	0.0239 (16)	0.0221 (16)	0.0198 (14)	−0.0062 (13)	0.0017 (11)	−0.0017 (11)
N2	0.0179 (14)	0.0235 (16)	0.0154 (13)	−0.0038 (12)	−0.0021 (10)	0.0026 (11)
N3	0.0275 (17)	0.0184 (16)	0.0251 (15)	−0.0027 (13)	0.0083 (12)	0.0011 (12)
C1	0.031 (2)	0.022 (2)	0.0214 (17)	0.0031 (16)	0.0031 (14)	−0.0010 (14)
C2	0.0229 (19)	0.036 (2)	0.0215 (17)	0.0055 (16)	0.0056 (14)	−0.0044 (15)
C3	0.0238 (19)	0.025 (2)	0.034 (2)	0.0090 (16)	0.0073 (15)	0.0025 (15)
C4	0.029 (2)	0.027 (2)	0.0198 (17)	−0.0050 (16)	0.0076 (14)	−0.0073 (14)
C5	0.0273 (19)	0.0212 (19)	0.0222 (17)	−0.0050 (15)	0.0056 (14)	−0.0048 (13)
C6	0.043 (2)	0.024 (2)	0.039 (2)	−0.0082 (18)	0.0167 (18)	0.0084 (16)
C7	0.0218 (18)	0.0180 (18)	0.0242 (17)	0.0023 (14)	0.0062 (13)	0.0017 (13)
C8	0.0184 (17)	0.025 (2)	0.0285 (18)	−0.0033 (15)	0.0049 (14)	−0.0017 (14)
C9	0.030 (2)	0.030 (2)	0.050 (3)	−0.0106 (18)	0.0016 (18)	−0.0093 (18)

### Geometric parameters (Å, º)

**Table:**

Sn1—O1^i^	2.076 (2)	C1—H1B	0.9800
Sn1—O1	2.106 (2)	C1—H1C	0.9800
Sn1—C1	2.114 (4)	C2—H2A	0.9800
Sn1—C2	2.121 (4)	C2—H2B	0.9800
Sn1—O2^i^	2.250 (2)	C2—H2C	0.9800
Sn1—N1	2.641 (3)	C3—H3A	0.9800
Sn2—O1	2.013 (2)	C3—H3B	0.9800
Sn2—C4	2.105 (3)	C3—H3C	0.9800
Sn2—C3	2.114 (4)	C4—H4A	0.9800
Sn2—O2	2.140 (2)	C4—H4B	0.9800
Sn2—N2	2.240 (3)	C4—H4C	0.9800
S1—C7	1.695 (4)	C5—H5	0.9500
O1—Sn1^i^	2.076 (2)	C6—H6A	0.9800
O2—C8	1.418 (4)	C6—H6B	0.9800
O2—Sn1^i^	2.250 (2)	C6—H6C	0.9800
N1—C5	1.297 (5)	C8—C9	1.503 (6)
N1—N2	1.393 (4)	C8—H8A	0.9900
N2—C7	1.334 (4)	C8—H8B	0.9900
N3—C5	1.353 (4)	C9—H9A	0.9800
N3—C7	1.371 (5)	C9—H9B	0.9800
N3—C6	1.452 (5)	C9—H9C	0.9800
C1—H1A	0.9800		
			
O1^i^—Sn1—O1	74.55 (9)	H1A—C1—H1C	109.5
O1^i^—Sn1—C1	106.36 (12)	H1B—C1—H1C	109.5
O1—Sn1—C1	99.22 (12)	Sn1—C2—H2A	109.5
O1^i^—Sn1—C2	106.31 (13)	Sn1—C2—H2B	109.5
O1—Sn1—C2	101.36 (12)	H2A—C2—H2B	109.5
C1—Sn1—C2	144.96 (16)	Sn1—C2—H2C	109.5
O1^i^—Sn1—O2^i^	70.95 (8)	H2A—C2—H2C	109.5
O1—Sn1—O2^i^	145.49 (9)	H2B—C2—H2C	109.5
C1—Sn1—O2^i^	90.83 (12)	Sn2—C3—H3A	109.5
C2—Sn1—O2^i^	88.04 (12)	Sn2—C3—H3B	109.5
O1^i^—Sn1—N1	148.36 (9)	H3A—C3—H3B	109.5
O1—Sn1—N1	73.83 (9)	Sn2—C3—H3C	109.5
C1—Sn1—N1	79.92 (12)	H3A—C3—H3C	109.5
C2—Sn1—N1	79.00 (12)	H3B—C3—H3C	109.5
O2^i^—Sn1—N1	140.66 (9)	Sn2—C4—H4A	109.5
O1—Sn2—C4	118.29 (13)	Sn2—C4—H4B	109.5
O1—Sn2—C3	114.01 (12)	H4A—C4—H4B	109.5
C4—Sn2—C3	127.46 (15)	Sn2—C4—H4C	109.5
O1—Sn2—O2	74.44 (9)	H4A—C4—H4C	109.5
C4—Sn2—O2	93.32 (12)	H4B—C4—H4C	109.5
C3—Sn2—O2	95.86 (13)	N1—C5—N3	111.5 (3)
O1—Sn2—N2	82.92 (9)	N1—C5—H5	124.3
C4—Sn2—N2	95.66 (13)	N3—C5—H5	124.3
C3—Sn2—N2	95.15 (14)	N3—C6—H6A	109.5
O2—Sn2—N2	157.27 (9)	N3—C6—H6B	109.5
Sn2—O1—Sn1^i^	112.77 (11)	H6A—C6—H6B	109.5
Sn2—O1—Sn1	141.62 (12)	N3—C6—H6C	109.5
Sn1^i^—O1—Sn1	105.45 (9)	H6A—C6—H6C	109.5
C8—O2—Sn2	125.4 (2)	H6B—C6—H6C	109.5
C8—O2—Sn1^i^	132.4 (2)	N2—C7—N3	106.8 (3)
Sn2—O2—Sn1^i^	101.70 (9)	N2—C7—S1	126.7 (3)
C5—N1—N2	105.8 (3)	N3—C7—S1	126.4 (3)
C5—N1—Sn1	136.2 (2)	O2—C8—C9	111.7 (3)
N2—N1—Sn1	117.7 (2)	O2—C8—H8A	109.3
C7—N2—N1	109.2 (3)	C9—C8—H8A	109.3
C7—N2—Sn2	127.3 (2)	O2—C8—H8B	109.3
N1—N2—Sn2	123.4 (2)	C9—C8—H8B	109.3
C5—N3—C7	106.7 (3)	H8A—C8—H8B	108.0
C5—N3—C6	128.2 (3)	C8—C9—H9A	109.5
C7—N3—C6	125.1 (3)	C8—C9—H9B	109.5
Sn1—C1—H1A	109.5	H9A—C9—H9B	109.5
Sn1—C1—H1B	109.5	C8—C9—H9C	109.5
H1A—C1—H1B	109.5	H9A—C9—H9C	109.5
Sn1—C1—H1C	109.5	H9B—C9—H9C	109.5
			
C4—Sn2—O1—Sn1^i^	88.55 (16)	O1^i^—Sn1—N1—N2	−9.0 (3)
C3—Sn2—O1—Sn1^i^	−86.31 (16)	O1—Sn1—N1—N2	−6.9 (2)
O2—Sn2—O1—Sn1^i^	3.23 (10)	C1—Sn1—N1—N2	95.9 (3)
N2—Sn2—O1—Sn1^i^	−178.81 (13)	C2—Sn1—N1—N2	−112.3 (3)
C4—Sn2—O1—Sn1	−96.9 (2)	O2^i^—Sn1—N1—N2	174.7 (2)
C3—Sn2—O1—Sn1	88.2 (2)	C5—N1—N2—C7	−1.2 (4)
O2—Sn2—O1—Sn1	177.8 (2)	Sn1—N1—N2—C7	−176.1 (2)
N2—Sn2—O1—Sn1	−4.3 (2)	C5—N1—N2—Sn2	−177.7 (2)
O1^i^—Sn1—O1—Sn2	−174.8 (3)	Sn1—N1—N2—Sn2	7.5 (3)
C1—Sn1—O1—Sn2	−70.2 (2)	O1—Sn2—N2—C7	−179.2 (3)
C2—Sn1—O1—Sn2	81.3 (2)	C4—Sn2—N2—C7	−61.3 (3)
O2^i^—Sn1—O1—Sn2	−175.41 (15)	C3—Sn2—N2—C7	67.2 (3)
N1—Sn1—O1—Sn2	6.38 (19)	O2—Sn2—N2—C7	−174.1 (3)
O1^i^—Sn1—O1—Sn1^i^	0.0	O1—Sn2—N2—N1	−3.4 (3)
C1—Sn1—O1—Sn1^i^	104.54 (13)	C4—Sn2—N2—N1	114.5 (3)
C2—Sn1—O1—Sn1^i^	−103.98 (14)	C3—Sn2—N2—N1	−117.0 (3)
O2^i^—Sn1—O1—Sn1^i^	−0.7 (2)	O2—Sn2—N2—N1	1.7 (4)
N1—Sn1—O1—Sn1^i^	−178.86 (13)	N2—N1—C5—N3	0.3 (4)
O1—Sn2—O2—C8	−175.8 (3)	Sn1—N1—C5—N3	173.6 (2)
C4—Sn2—O2—C8	65.7 (3)	C7—N3—C5—N1	0.7 (4)
C3—Sn2—O2—C8	−62.5 (3)	C6—N3—C5—N1	−178.0 (4)
N2—Sn2—O2—C8	178.9 (3)	N1—N2—C7—N3	1.7 (4)
O1—Sn2—O2—Sn1^i^	−2.81 (9)	Sn2—N2—C7—N3	177.9 (2)
C4—Sn2—O2—Sn1^i^	−121.28 (13)	N1—N2—C7—S1	−177.6 (3)
C3—Sn2—O2—Sn1^i^	110.52 (13)	Sn2—N2—C7—S1	−1.3 (5)
N2—Sn2—O2—Sn1^i^	−8.1 (3)	C5—N3—C7—N2	−1.5 (4)
O1^i^—Sn1—N1—C5	178.2 (3)	C6—N3—C7—N2	177.3 (3)
O1—Sn1—N1—C5	−179.7 (4)	C5—N3—C7—S1	177.8 (3)
C1—Sn1—N1—C5	−76.9 (4)	C6—N3—C7—S1	−3.4 (5)
C2—Sn1—N1—C5	75.0 (4)	Sn2—O2—C8—C9	−112.7 (3)
O2^i^—Sn1—N1—C5	1.9 (4)	Sn1^i^—O2—C8—C9	76.5 (4)

Symmetry code: (i) −*x*+1, −*y*+1, −*z*+1.
